# Network characteristics emerging from agent interactions in balanced distributed system

**DOI:** 10.1186/s40649-015-0019-2

**Published:** 2015-07-15

**Authors:** Mahdi Abed Salman, Cyrille Bertelle, Eric Sanlaville

**Affiliations:** LITIS, Normandy University, Le Havre, France

**Keywords:** Complex networks, Network structure, Distributed computing systems: Information spreading, Load balancing, Multi-agent simulation

## Abstract

A distributed computing system behaves like a complex network, the interactions between nodes being essential information exchanges and migrations of jobs or services to execute. These actions are performed by software agents, which behave like the members of social networks, cooperating and competing to obtain knowledge and services. The load balancing consists in distributing the load evenly between system nodes. It aims at enhancing the resource usage. A load balancing strategy specifies scenarios for the cooperation. Its efficiency depends on quantity, accuracy, and distribution of available information. Nevertheless, the distribution of information on the nodes, together with the initial network structure, may create different logical network structures. In this paper, different load balancing strategies are tested on different network structures using a simulation. The four tested strategies are able to distribute evenly the load so that the system reaches a steady state (the mean response time of the jobs is constant), but it is shown that a given strategy indeed behaves differently according to structural parameters and information spreading. Such a study, devoted to distributed computing systems (DCSs), can be useful to understand and drive the behavior of other complex systems.

## Introduction

In a complex system, individual nodes (or agents, or actors) take individual decisions depending on the information they can retrieve from other nodes. The global behavior of the system cannot be predicted by these individual decisions alone, as they produce complex interactions. For a better understanding, simulation is often the best way.

A distributed computing system (DCS) is a complex system because it is composed of a set of computing nodes connected by a communication network, and each node (in fact, the software agent(s) that is (are) hosted in the node) takes its own decisions. The global “purpose” of the DCS is to perform a high number of jobs or services, but there is no central authority to distribute these tasks to the nodes. Each task (hereafter called a job for simplicity) is initially proposed to one given node. That is why in DCSs, the interactions between components take two forms: communication between nodes to know each other’s load (the resource discovery phase) and migration of jobs between nodes (the load balancing phase). In a DCS, load balancing aims at enhancing the resource usage. It tries to distribute the load (that is, jobs to process) evenly between system nodes and to minimize the mean job response time. The global behavior of the system is monitored by computing the mean response time of the jobs, the distribution of the load (usually the size of the job queue for each node), and the amount of migrations. The aim is to maintain the system in an equilibrium state (a steady state), with low operating costs.

Note that in most applications of complex systems, especially on networks involving people, one may identify this first communication phase, followed by a second phase where services, or resources, are shared (not always respecting fairness) between the members of the network. Newman [1] gave a survey on many types of real-world networks, including social network modeling for instance business relationships. See also [2] about job information networks and [3] for a more economic point of view on social networks.

Most works in the DCS literature investigate the two phases separately. Some works related to these two research fields are given below. However, we study in this paper the impact of the structure of the network resulting from resource discovery methods on the performance of a load balancing strategy.

### Resource discovery

For each node, the knowledge of other node states is essential for cooperation purpose. In particular, the efficiency of load balancing depends on quantity, accuracy, and distribution of available information [4]. Information is either obtained directly by querying neighbor nodes or provided by a more sophisticated resource discovery method [5].

The usual objective is to minimize the quantity of collected information while retaining an optimal performance for load balancing. Indeed, decreasing the quantity of required information at nodes will decrease the search space and communication complexity. However, *different distributions of information between nodes will produce different structures for the resulting network* (called overlay network in the paper): this non-physical network keeps track of the knowledge at each node, at each time. Its structure depends both on the initial, physical network and on the resource discovery method. It has an impact on the load balancing strategies’ efficiency, hence on the global system performance.

Volgaris et al. [6] presented the Newscast model, which is “an epidemic protocol for disseminating information in large, dynamically changing sets of autonomous agents.” Authors showed that the snapshots of an overlay network (they called it series of the communication graphs) of this model exhibit stable small-world properties. These properties are not intended or expressed explicitly by agent design, but they are emergent from the underlying simple epidemic-style information exchange protocol.

### Load balancing

Load balancing strategies specify scenarios of cooperation between nodes. In most DCSs, load balancing takes place exclusively among a few neighbor nodes (that is, directly connected nodes) and, hopefully, a global equilibrium is achieved. In this paper, mechanisms that are more sophisticated are considered.

Willebeek-LeMair and Reeves [4] proposed five load balancing schemes. However, only receiver-initiated diffusion (RID) and sender-initiated diffusion (SID) schemes are using local knowledge. Nodes frequently broadcast their current load status to all of their direct neighbors. In SID, a heavily loaded node initiates a migration towards nodes whose load is below a threshold. In RID, a node whose load drops below a threshold requests a migration from all its direct neighbors, which are overloaded. Cao et al. [7] presented a load balancing framework called mobile agent-based load balancing (MALD) that uses stationary agents that monitor the workload on local servers and mobile agent to carry loads to underloaded server. Hence, this is an SID scheme but controlled by agents.

Fukuda et al. [8] analyzed the effectiveness of using statistical properties of the network structure in multi-agent systems. They dealt with the problem of server agent deployment and server selection by client agents in the internet. Authors showed that the scale-free characteristics and degree distribution of the network play an essential role in the performance of the studied algorithm. Although their problem is different from ours, the underlying ideas are similar.

Laredo et al. [9] presented an online and decentralized scheduler (in fact, a load balancing scheme) based on a self-organized criticality model classically called *sandpile*. Authors show that a sandpile model [10] yields a better performance if the nodes are arranged as a small-world network rather than a lattice 2D grid. The sandpile model is further analyzed in this paper.

In [11], a new load balancing strategy for distributed computing system has been adapted from the RID scheme (but it selects the migration source node). It is called HLM for help local maximum. A comparison of performances has been made between HLM, the SID model proposed in [4], and the sandpile scheduler proposed in [9]. The former outperforms the two other strategies when the network exhibits a small-world structure. In [12], the impact of network structure on the behavior of load balancing strategies is investigated. Authors showed that this structure often has the same (to some extent) effect on the job mean response time whatever the load balancing strategy is used. In this paper, we extend the work in [12] through adding new mechanism of interaction and cooperation between system agents.

The remaining of the paper is organized as follows: the “Network structures” section briefly reviews network models used by information exchange. The “Resource discovery and the overlay network” section sketches the three resource discovery methods that are tested here. The main strategies of load balancing are explained in the “Load balancing strategies” section. A new mechanism of interaction and cooperation between system agents is presented in the “Improving information management” section. The “Agent-based simulation” section presents the simulation, parameters, and obtained results. The “Conclusion” section discuses these results.

## Network structures

A network is a set of entities that are linked by a given relation [1]. For purposes of analysis and development, a network is modeled mathematically using graphs. Graph theory is the most important mathematical technique used to model the geomorphological relations among the entities in a system. Nodes represent entities. Links (edges or arcs) connect nodes to show an existing relation between them.

A graph is denoted by *G*=(*V*,*E*) where *V* is the set of vertices and *E* is the set of edges (undirected links). Two vertices *u*,*v*∈*V* are neighbors if and only if (*u*,*v*)∈*E*.

A directed graph (called digraph) is denoted by *G*=(*V*,*A*), where *A* is the set of arcs (directed links). For any arc (*i*,*j*), *i* and *j* are its source and target, respectively. *i* is called the ancestor (predecessor) of *j*, and *j* is the successor of *i*. However, in each domain, the conceptual pair (vertex, link) is redefined. For example, in the web, vertices are web pages and links are hyperlinks; in the Internet, vertices are autonomous systems and links are Internet connectivity relationships; and in the social networks, vertices are the population members (individuals or organizations) and links represent acquaintance (conceptually called a friendship). DCSs like most complex networks are modeled by graphs, directed or not, whose vertices are the components or nodes, and edges or arcs capture the interactions between components. A network can be physical or logical (depending on how an edge is defined) and static or dynamic (depending on edge stability with respect to time). When a DCS system is started, either each node discovers its environment using some search mechanisms or the system administrator initially provides each one with a small set of links. Thus, each node has from the start a set of neighbors. Such network is called *the initial or underlay network*. A node only knows its neighbors and their characteristics (like computing resources, load, etc.).

The network is static when each node keeps the same set of neighbors during the system life and dynamic when sophisticated resource discovery methods are used: each cycle, a node exchanges information with its direct neighbors about the status of other nodes. A system may use both network types, especially when authentication is necessary for communication. In such cases, information broadcasting creates what is called an *overlay* network from the initial network. In peer-to-peer networks for instance, an overlay network is a logical network that is built from the information exchanged between peers using some diffusion method. It is modeled by a directed graph, where an arc (*i*,*j*) means that node *i* has some knowledge about node *j*. In this paper, the same model is used, the exchanged information being the load of some nodes.

The impact of both types of network structure on the performance of several load balancing strategies is studied here using a simulation. The structure of networks that emerges from interaction between nodes is described. Initial networks are generated using theoretical models. Five models have been chosen for this study. Below, we give a brief description of their characteristics and the way they are generated.

### Graph models

#### Barabàsi–Albert

The model of Barabàsi and Albert [13] creates random scale-free networks using a preferential attachment mechanism. Such networks admit a power law (or scale-free) degree distribution for their nodes. They are created using two important general concepts: growth and preferential attachment. Growth means the nodes are added one after the other. Preferential attachment means that new nodes prefer to attach themselves to highly connected older nodes in the network.

#### Random graph

Different models were introduced according to the definition of randomness. The most common Edgar Gilbert model [14] imposes that a graph *G* of *n* nodes is generated by adding each edge with independent probability 0<*p*<1. In Erdös and Rényi model [15], a graph with *N* nodes and *M* edges is chosen uniformly at random from all possible graphs of same *N* and *M*. The latter is a general model for any graph. The former is used in this paper to generate random graph.

#### Random Euclidean

In this model, Cartesian coordinates are assigned randomly on a plane to each node. An edge is created between any two nodes if the Euclidean distance between them is less than a given threshold. The same structure is produced but results in a smaller diameter when nodes are distributed on a *sphere*. It is called Euclidean sphere in this paper. A small threshold will often result in disconnected graphs.

#### Watts–Strogatz

The Watts–Strogatz model generates a random graph with small-world properties, i.e., short average path lengths and high clustering index. The mean degree of the nodes is rather small but the distance between any two randomly chosen nodes is proportional to the logarithm of graph order. This model is very popular in complex network studies since Duncan J. Watts and Steven Strogatz proposed it in 1998 in *Nature* [16].

### The different uses of the graph models

Each of the chosen models shows a specific combination of characteristics. These characteristics are present in different types of realistic complex networks.

The Barabàsi–Albert model is characterized by the features of scale-free and preferential attachment. Many real networks like the web [13], the Internet [17], and some social networks [18] exhibit these features. Random graphs are a general model that can be used as a reference for most real network types. Furthermore, a range of complex networks share features of random graphs [19]. Random Euclidean graphs have a relatively long diameter but high clustering coefficient. Random Euclidean graphs are used to model type networks when node attributes include spatial information, as in the modeling of ad hoc wireless networks [20].

Finally, the Watts–Strogatz model is characterized by the small-world phenomena. Any node is reachable from anywhere in the graph with a few number of traversed edges. It also shows a large clustering coefficient. The information network web and other real networks have that small-world feature [1, 16, 21]. The Watts–Strogatz, random, and scale-free models are used in [22] to evaluate knowledge sharing in social commerce using an agent-based computational approach.

## Resource discovery and the overlay network

Three schemes are tested for information collecting during each cycle: local, rumor spreading, and mobile agents. In local scheme, a node asks all its direct neighbors about their load status. The initial and the overlay networks are the same throughout the life of the system. The two other methods build dynamic overlay network and are called global schemes throughout the paper. In rumor spreading (see [23–25]), a node chooses one of its direct neighbors at random and exchanges available information from recent cycles. If a node just sends information, it is called a *PUSH* protocol, a *PULL* protocol is considered when a node just receives information, and a mixed scheme (considered in this study) does both actions (PUSH–PULL) each cycle. In mobile agent-based broadcasting method (for more details, see for example [26]), roaming objects (called mobile agents) visit nodes and exchange valid information with them. A mobile agent chooses the next destination from one of the current node’s direct neighbors at random or using a specific transfer mechanism. In both latter cases, a node may get information from nodes located in a distance equal to the specified TTL (time-to-live) limit. Collected information is stored locally in a table with limited capacity (and the information is kept at most until its TTL is reached). Another important feature is that in rumor spreading as in mobile agent-based method, information travels through the *underlay* network.

While the information on node *j* is kept in the table of node *i*, the arc (*i*,*j*) exists in the directed graph which represents the *overlay* network. Hence, the overlay network is highly dynamic since arcs are frequently replaced. Indeed, many parameters affect the structure of the resulting network. The first parameter is the underlay network structure, because, as already stated, information uses this network to spread. The second parameter is the TTL. The greater the value of the TTL, the smaller the diameter of the overlay network, but gathered information becomes less accurate. Another parameter is the capacity of local caches (or equivalently, the size of the table). Its limit restricts the maximum out-degree of the resulting network. When TTL is high and cache capacity is small, only the most recent *k* bits of information are kept (*k* is the capacity of local cache) and extra information is dropped.

Nodes can use this information on the load distribution to decide of job migrations. The different load balancing strategies are presented in the next section. Many tests are done to show the impact of the underlay network, TTL, cache capacity, and broadcasting method parameters on the final structure of the overlay network; the ‘Results’ section displays some of their results.

## Load balancing strategies

Load balancing in DCSs depends on many parameters, which make it a complex problem. Hence, load metrics should express the authentic state of a node. *In this paper, the load is measured by the remaining time of jobs being executed, plus the execution times of waiting jobs.*

A strategy should specify four policies [4]:

1. Information: when and how to collect information (see the “Resource discovery and the overlay network” section).
2. Initiation: who triggers the load migration process?
3. Source and destination: when the decision is taken to move some load, characteristics of source (among overloaded nodes) and of destination (among underloaded ones) should be specified.
4. Load selection: determines the properties of the load that is more suitable to be migrated to the destination node.

Four strategies are tested in this paper. They differ either in policy 2, 3, or both. The other policies are the same for all tested strategies. A node migrates at most one job each cycle. Hence, strategies are adapted to this constraint. The names and the policies of tested strategies are given below.

Note that in these descriptions, the neighborhood of a node *i* is the set of nodes whose load is known by *i* (that is, the neighbors of *i* in the overlay network at this cycle).

### SID

Any overloaded node initiates the migration process. It chooses randomly one underloaded node as destination from its neighborhood [4]. Hence, in each cycle, an overloaded node can send only one job but an underloaded one may receive several jobs from different nodes.

### RID

An underloaded node looks in its neighborhood for overloaded nodes to migrate loads from. A possible source node is chosen at random. Hence, in each cycle, the initiator can receive only one job while the sender may send several jobs to different nodes.

### Sandpile

The load of a given node is avalanched (dropped) down to some neighbor nodes, if some criteria are met. For example, in [9], a node chooses two neighbor nodes at random. The load is distributed evenly among the three nodes when the load of the current node is greater than the summation of the two. Hence, an overloaded node may send several jobs to its two neighbors. One migration in sandpile may trigger other migrations in a cascading way until no migration is possible any more. Hence, the network should reach an equilibrium state each cycle (in this study, a node is inspected only once at each cycle).

### HLM

This strategy is RID, except that an underloaded node demands a migration from the maximum loaded node in its neighborhood. Hence, a heavily loaded node may respond to many requests of migrations during one cycle.

A node is considered overloaded or underloaded according to the average load of its neighborhood. Nodes in a neighborhood are classified into three categories: overloaded, intermediate, and underloaded. Intermediate nodes do not take part in the load balancing process (except possibly as receiver in sandpile strategy).

Once the source and destination are specified by process trigger, the latest arrived job for source node is prepared for migration. Note that other job selection policies are applicable, like earliest arrived, shortest execution, etc.

The performance of each load balancing strategy is evaluated using the mean response time (MRT) criterion. The response time of a job is the duration from its time of submission to its completion time. MRT is computed on all jobs processed in a given interval, here, the total simulation time.

## Improving information management

Load balancing depends on information provided by resource discovery. Load balancing may also participate in spreading information by means of migration dialog. Hence, some mechanism of local interaction between information management and the migration process is added. This interaction changes the overlay structure and enhances obtained results.

### Reinforcement

A node initiates load balancing with another node that is chosen from local cache according to selection policy. Actually, the node uses uncertain information, since the cache may have items that have age greater than one, i.e., up-to-date. The initiator starts communication with selected partner by asking for its current state. It adds received information to the cache with age=0. This “fresh” information is spread through the network. As a result, candidate partners become known in area out of TTL distance with up-to-date information and may be selected by more nodes.

### Information preference

When the number of collected items is larger than cache size, extra entries need to drop. Dropping is made after sorting entries by some preferred order. By default, cache items are ordered by their recentness, i.e., their age.

Load balancing prefers nodes that succeeded in most previous migration processes. Hence, load balancing sets a variable called *activeness* that is associated with node’s descriptor fields. Activeness value equals the difference between the number of sent jobs and number of received jobs.

After dropping outdated items from the cache, a node sorts the remaining items by activeness in ascending or descending order according to whether a sender- or receiver-initiated diffusion load balancing is used. SID uses ascending order, and RID uses descending order. Then, it drops any extra entries according to that sorting.

## Agent-based simulation

A simulation program has been designed using Java language based on the GraphStream package [27]. The developed package provides an easy-to-use library of generators and methods for dynamic graphs. A node class has been extended to facilitate agent’s management. Agents are autonomous objects that take decisions according to their internal state and/or environment state. An agent interacts with other agents to accomplish specific tasks. Agents have been frequently used to simulate various complex systems [28, 29]. In social networks, agent-based models can be used to simulate real actors [22].

### Agent modeling

In our model, a node *v* hosts three types of stationary agents. One of them optionally receives and hosts mobile agents. Below, definitions and assigned tasks for each agent type are presented:

The scheduler, *S*, is responsible for receiving arrived jobs and schedule them for execution or migration. An arrived job has to wait in a queue when some resource currently is not available. *S* uses FCFS (first come first serve) policy to execute arrived jobs.

The information manager, *I*, is responsible for communicating and exchanging information with neighbor nodes in the underlay network. *I* applies information collection policy and runs selected resource discovery method, see the “Load balancing strategies” and “Resource discovery and the overlay network” sections. Load information is cached in a list *D*. An entry *d*∈*D* is dropped when its age exceeds predefined time-to-live limit denoted by TTL.

The balancing agent, *B*, applies a selected load balancing strategy. *B* uses local information that is maintained by *I* to trigger load balancing process. It determines the source and destination nodes for the migration from content of *D*. An initiator *B* contacts the corresponding *B* at another node to move one job between them.

A mobile agent (when used) *M* is a roaming object. *M* moves from node to node like a bee. *I* usually welcomes *M* and exchanges information with it. *M* transfers itself to one of the current *I*’s neighbors that is chosen at random.

### Parameter settings

Instances of the initial network are obtained using GraphStream generators. All networks have 1024 nodes. Table 1 shows the features of the network instances that have been tested on a laptop.

**Table 1:** Network instances

Model	*d*(*G*)	*Δ*(*G*)	*δ*(*G*)	$\overline {C}$
Barabàsi–Albert	5	95	4	0.030
	4	149	8	0.059
	4	175	12	0.070
	3	210	16	0.088
	3	258	24	0.188
Random graph	6	19	1	0.008
	4	30	5	0.015
	4	38	9	0.023
	3	55	18	0.031
	3	66	29	0.047
Random Euclidean	36	19	1	0.595
	23	30	1	0.611
	18	41	6	0.621
	15	53	12	0.620
	12	76	12	0.630
Euclidean sphere	31	18	1	0.590
	16	30	5	0.590
	12	39	10	0.592
	11	47	12	0.595
	8	66	30	0.591
Watts–Strogatz	9	12	5	0.484
	6	20	12	0.525
	5	29	19	0.521
	4	38	25	0.538
	4	57	42	0.535

Five networks of different densities are generated for each model. The average degrees are 8, 16, 24, 32, and 48. *d*(*G*) is the network diameter, *Δ*(*G*) is the maximum node degree, *δ*(*G*) is the minimum node degree, and $\overline {C}$ is the average clustering coefficient of a graph. The clustering coefficient of the node *v* is the density of a sub-graph that is composed only of *v* neighbors. Density is the ratio of the number of edges over the maximum number of edges in a graph of the same order, i.e., $\frac {\mid E \mid }{N(N-1)}$ (*E* is the edge set and *N* is the number of nodes).

Jobs arrive to nodes directly according to a Poisson process. Analyses of traces of production systems are carried in [30–32]. Authors showed that the most important cycle noticed in job arrival distribution is the daily cycle. Basically, our simulation spans a duration of 1 day. In simulation, a time unit is called *cycle* or *round* (we use the former). To reduce computation, a cycle is considered equal to 1 min, i.e., at minimum, our simulation spans 1440 cycles.

Jobs arrive to nodes directly according to the local arrival rates (that is, rates differ from one node to another) that are generated uniformly *with mean and standard deviation equal to 1*. A workload instance is generated using the models proposed in [30]. According to arrival rate distribution, nodes vary in number of jobs that they receive per cycle. The node with maximum arrival rate receives ≃2 jobs per cycle. Without migration, that node would complete the execution of the last received job at time approximately equal to the doubled submission time.

The workload instance contains 1,474,560 jobs. Trace analyses of DCSs show that many jobs have small duration (except for a few very large ones). Following [9], we considered only jobs of duration equal to one cycle. Note that additional experiments showed that different durations do not significantly change our results.

Laredo et al. [9], Cao et al. [7], and others migrate jobs between nodes instantly. We considered that migration of a job from one node to another takes one cycle.

A node classifies entries of local cache into 40 % underloaded, 20 % intermediate, and 40 % overloaded. It compares its own load and takes decision of participating in load balancing according to the selected strategy.

First, all strategies are applied on static networks, i.e., local scheme of information collection is used. We compute the performance (measured by the MRT on all jobs) of each strategy for different parameter settings. The number of tests is 100=(4 strategies × 25 network instances).

Then, the characteristics are computed for the overlay networks resulting from the two global discovery methods, 25 network instances, and TTL with values 1,2,3,4, and 5. No limit is specified for local cache in these tests.

For each run, the diameter, average clustering coefficient, and average out-degree of resulting overlay networks are computed (using a snapshot of the overlay network taken when the structure becomes stable. The number of tests is 250=(2 methods × 25 networks ×5 TTL).

Finally, the performance of each load balancing strategy is computed using overlay networks. The two discovery methods are used on all initial networks. The other parameters are as follows: TTL is 5 and cache size is chosen to be 8, 16, 24, 32, and 48.

The number of tests is 1000=(2 methods × 4 strategies × 25 network instances × 5 sizes of cache).

### Results

When no migration is enabled, no equilibrium state is reached and the MRT keeps increasing. With load balancing, in all tests but a very few (see below), convergence towards a steady state is achieved in less than 200 cycles. Due to the large number of results, only few figures are presented.

#### Local scheme

**Fig. 1:**
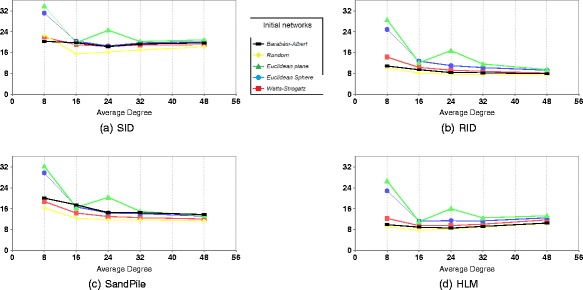
Mean response time for different network models: local scheme of resource discovery. **a** SID. **b** RID. **c** Sandpile. **d** HLM

Figure 1 shows the mean response time (MRT) computed during the simulation time of 1440 cycles. The figure includes results of four strategies. The tests use local scheme of resource discovery. Each curve represents a model of the initial network. The *X*-axis gives the average degree of the initial network.

The figures show that the impact of network structure differs from one strategy to another in values only. The network density plays a noticeable role in enhancing the performance of all strategies. In particular, for random Euclidean graphs, no steady state is reached when the average degree is 8.

The same pattern of MRT for each network model has been shown for all strategies. The performance of SID, RID, and Sandpile is enhanced whenever the average degree is increased. This is due to random selection of source or destination node.

HLM has another behavior, its performance enhances until average degree reaches 24 (the load of heavily loaded nodes decreases rapidly), then it is degraded, since the number of possible sources (maximum nodes in each neighborhood) decreases when the neighborhood size increases (as many overloaded nodes are not selected as sources).

For all strategies, the preference of network models is ordered as random, Barabàsi–Albert, Watts–Strogatz, random Euclidean sphere, and random Euclidean plane, which is the same order as the order of their diameters.

The results of the applied strategy on networks of the same average degree vary according to the network diameter. The smaller the diameter, the better the MRT. This is clear in the difference of performance using random Euclidean on plane and random Euclidean on sphere since they differ only in their diameter.

#### Global scheme

**Fig. 2:**
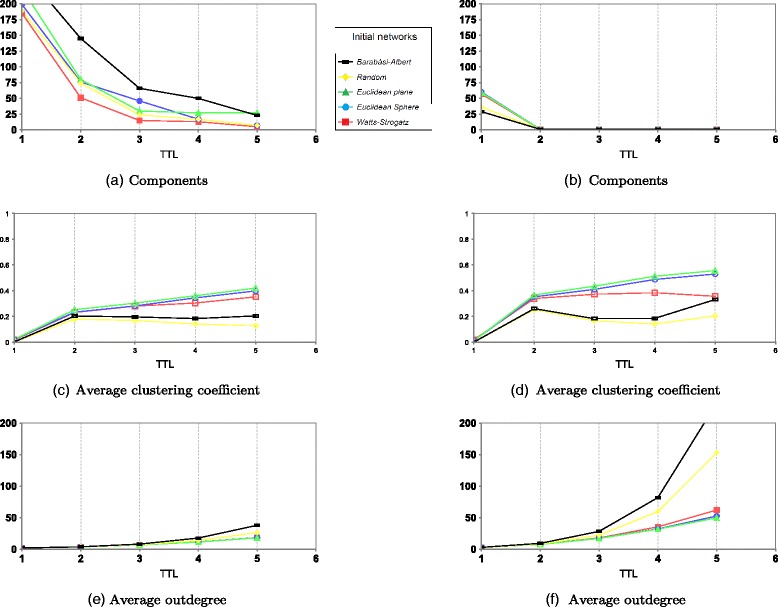
Overlay network features: *left* from mobile agent and *right* from rumor spreading. **a**, **b** Components. **c**, **d** Average clustering coefficient. **e**, **f** Average out-degree

The average degree of an overlay network resulting from rumor spreading or mobile agent resource discovery methods depends on the TTL value. Figure 2 shows the number of components, average clustering coefficient $\overline {C}$, and average degree of obtained overlay networks from both methods. Low TTL values give non-connected graphs.

Mobile agent-based discovery method differs from rumor spreading: sometimes, nodes may not be visited by mobile agent, while in rumor spreading-based method, a node is always concerned with one exchange at least. Hence, the overly network may remain disconnected for values of TTL<5 or if the initial network is Barabàsi–Albert (see Fig. 2 a).

Tables 2 and 3 show the MRT of four load balancing strategies using mobile agent and rumor spreading discovery methods, respectively. The result is obtained using same cache size (16 entries) but different average degrees of initial networks. TTL=5 is used.

**Table 2:** MRT on each underlay network using mobile agent

	Barabási–Albert	Random	Euclidean plane	Euclidean sphere	Watts–Strogatz
SID					
8	15.533	15.362	15.635	15.482	15.15
16	15.366	15.098	15.157	15.137	15.095
24	15.426	15.051	15.576	14.905	15.025
32	15.549	15.003	14.983	15.096	15.008
48	15.489	14.913	15.122	14.992	15.095
RID					
8	8.459	8.402	8.778	8.581	8.466
16	8.523	8.43	8.498	8.508	8.438
24	8.594	8.419	8.701	8.508	8.452
32	8.607	8.421	8.243	8.518	8.466
48	8.612	8.498	8.589	8.536	8.501
Sandpile					
8	14.517	14.095	14.939	14.352	13.915
16	14.781	14.025	14.239	14.256	13.962
24	14.848	13.973	14.826	14.151	13.966
32	15.129	14.106	14.039	14.378	13.982
48	15.215	14.075	14.425	14.296	14.098
HLM					
8	10.153	10.109	10.252	10.136	10.081
16	10.058	10.028	10.058	10.044	10.031
24	10	10.004	10.039	9.976	10.036
32	10.006	9.995	9.667	9.959	9.929
48	9.991	10.054	9.943	9.935	10.029

**Table 3:** MRT on each underlay network using rumor spreading

	Barabási–Albert	Random	Euclidean plane	Euclidean sphere	Watts–Strogatz
SID					
8	14.354	14.432	14.686	14.361	14.315
16	14.339	14.305	14.288	14.38	14.324
24	14.361	14.269	14.673	14.369	14.493
32	14.411	14.257	14.264	14.369	14.372
48	14.44	14.296	14.493	14.246	14.42
RID					
8	8.105	8.098	8.51	8.197	8.087
16	8.106	8.088	8.156	8.15	8.105
24	8.12	8.08	8.467	8.186	8.127
32	8.109	8.077	7.903	8.191	8.141
48	8.106	8.105	8.202	8.195	8.161
Sandpile					
8	12.985	12.924	14.255	13.378	12.923
16	13.085	12.84	13.233	13.215	13.14
24	13.115	12.924	13.991	13.247	13.12
32	13.203	13.02	13.258	13.336	13.139
48	13.287	13.075	13.336	13.34	13.1
HLM					
8	9.034	9.036	9.243	9.112	9.05
16	9.009	9.001	9.015	9.132	9.052
24	9.001	8.996	9.194	9.081	9.004
32	9.012	8.987	8.757	9.051	9.033
48	9.007	8.969	9.116	9.043	9.016

It is obvious from the tables that changing the average degree of the initial network has no much effect when the cache size is fixed. This is normal since load balancing depends on the structure of the overlay network in global scheme.

**Fig. 3:**
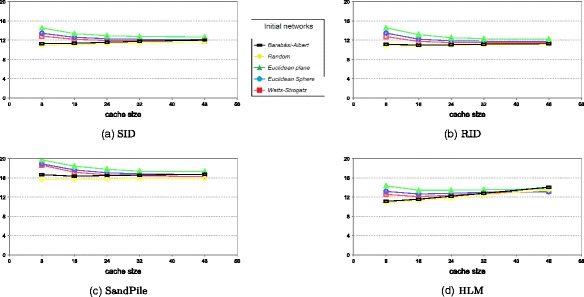
The impact of cache size on the MRT (average degree of initial networks = 8, rumor spreading, TTL = 5). **a** SID. **b** RID. **c** Sandpile. **d** HLM

This result is confirmed when using different cache sizes with same average degree of the initial network. The patterns are very similar to the ones observed for the local scheme (when considering the cache size instead of the average degree). Figure 3 shows the results obtained from using rumor spreading discovery method, different cache sizes, and same average degree of the initial network.

If we compare with the local scheme by considering cache size as the average degree of overlay network, the results of global scheme are much better.

#### Global scheme with improvement

The performance of load balancing is computed using three ways of information managements: the default one that is created by caching recent information, the second version where load balancing agent alters local cache by updating it with accurate information (reinforcement), and the last version where resource discovery agent filters cached entries according to their activeness values (information preference).

**Fig. 4:**
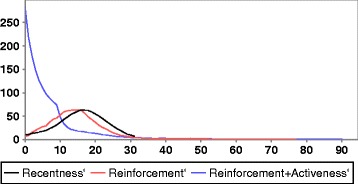
In-degree distribution of overlay networks

Figure 4 shows in-degree distributions of three snapshots of overlay network of three different experiments. Curves are smoothed by using moving average instead of real values. Black curve is obtained for an experiment in which cache items are ordered by recentness (the default method). It shows a small-world graph-like distribution. Red curve shows the use of reinforcement mechanism. That decreases the mean degree but stretches the tail of the curve. Reinforcement and activeness preference make the in-degree distribution be like one of a scale free (blue curve).

**Fig. 5:**
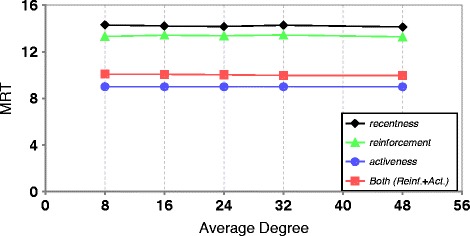
The impact of using cooperation mechanism on MRT values. Watts–Strogatz model, rumor spreading, SID load balancing strategy

Experiments are made using the proposed mechanism of cooperation between information manager *I* and load balancing responsible *B*. Figure 5 shows that the performance of load balancing is enhanced when applying the two techniques’ reinforcement and activeness. Using activeness indicator gives best result.

### Discussion

From the simulation, we may distinguish five main features:

- A steady state is always reachable (except for random Euclidean graphs with very small average degree and local scheme).
- With the global scheme, MRT is much better than with the local scheme because the overlay network is dynamic which let far nodes become neighbors at some moment or another.
- The best performance is made by the HLM load balancing strategy (RID is the closest one to HLM). However, MRT increases if the average degree or the cache size is large. That is because the same overloaded node is chosen by many nodes for migration.
- Rumor spreading-based resource discovery has better performance than mobile agent, but it demands more communications.
- The effect of the underlay network structure on the obtained MRT is more visible with the local scheme, especially for small average degree networks. Load balancing performs best on the underlay network of random or Watts–Strogatz model. In the global scheme, a small difference is noticed between the obtained MRT for different underlay network models that vanishes when increasing the average degree. The structure of the overlay network is different depending on cache management policy. It always has small-world features when the items of local cache are kept based on their recentness only. It is more scale free when activeness or reinforcement is used. In that case, MRT is enhanced for using reinforcement or/and activeness. Notice that the scale-free property is more helpful for the overlay than for the underlay networks. Indeed, nodes with the highest in-degree are also the most often selected for migration.

## Conclusion

In this study, the evolution of a complex system modeling distributed computer systems is simulated. The nodes of the DCSs have the resources to execute the jobs submitted to the system. The global objective of the system is to execute the jobs at a rate that matches their arrival rate. Nodes are associated to software agents that can take local decisions that guide the system behavior through two mechanisms: knowledge discovery (what is the workload of the other nodes?) and load balancing (if I am too much loaded, to which node can I send jobs?). In this paper, different methods are tested for these two mechanisms. The nodes are initially linked by a physical network, as the internet or a local network (the underlay graph). Knowledge discovery can be done by two ways: either locally, one node knows the load of its neighbors in the underlay graph, or globally, one node keeps information from a subset of nodes throughout the network (this knowledge is represented by the arcs of an overlay graph). Our tests show, as expected, that the performance of the global scheme is better in terms of response time. More importantly, the structure of the underlay network has little influence then; the overlay graph acquires a “small-world” structure (and, for some variants of the global scheme, a “scale-free” structure that is even more efficient). Four load balancing strategies were tested. All of them were able to keep the system in a steady state. However, strategies, which make use of all the information available, obtain a slightly better response time than others less sophisticated (and less complicated) like the Sandpile-based one.

The conclusions of this study may extend to other complex systems, especially social networks with information communication and the sharing (with or without fairness) of goods, resources, tasks, or jobs: when the knowledge discovery inside the network is efficient enough, the structure of the initial network is not important. Furthermore, the paper provides some insight on the way the sharing process (in our context, the load balancing) may be implemented to guarantee that the system reaches equilibrium.
